# Chronic kidney disease and the risk of cancer: an individual patient data meta-analysis of 32,057 participants from six prospective studies

**DOI:** 10.1186/s12885-016-2532-6

**Published:** 2016-07-16

**Authors:** Germaine Wong, Natalie Staplin, Jonathan Emberson, Colin Baigent, Robin Turner, John Chalmers, Sophia Zoungas, Carol Pollock, Bruce Cooper, David Harris, Jie Jin. Wang, Paul Mitchell, Richard Prince, Wai Hon. Lim, Joshua Lewis, Jeremy Chapman, Jonathan Craig

**Affiliations:** Sydney School of Public Health, University of Sydney, Sydney, Australia; Centre for Transplant and Renal Research, Westmead Hospital, Westmead, Australia; Clinical Trial Service Unit and Epidemiological Studies Unit, Nuffield Department of Population Health, Oxford, UK; Medical Research Council Population Health Research Unit, Nuffield Department of Population Health, Oxford, UK; School of Public Health and Community Medicine, University of New South Wales, Sydney, Australia; The George Institute for Global Health, Sydney, Australia; Faculty of Medicine, Nursing & Health Sciences, Monash University, Clayton, VIC Australia; Northern Clinical School, Kolling Institute of Medical Research, University of Sydney, Sydney, Australia; Centre for Vision Research, Westmead Millennium Institute of Medical Research, University of Sydney, Sydney, Australia; School of Medicine and Pharmacology, The University of Western Australia, Crawley, WA Australia

**Keywords:** Cancer epidemiology, Chronic kidney disease, Survival analyses

## Abstract

**Background:**

Chronic kidney disease (CKD) is an established risk factor for cardiovascular disease but the relevance of reduced kidney function to cancer risk is uncertain.

**Methods:**

Individual patient data were collected from six studies (32,057 participants); including one population-based cohort and five randomized controlled trials. Participants were grouped into one of five CKD categories (estimated glomerular filtration rate [eGFR] ≥75 mL/min/1.73 m^2^; eGFR ≥60 to <75 mL/min/1.73 m^2^; eGFR ≥45 to <60 mL/min/1.73 m^2^; eGFR <45 mL/min/1.73 m^2^; on dialysis). Stratified Cox regression was used to assess the impact of CKD category on cancer incidence and cancer death.

**Results:**

Over a follow-up period of 170,000 person-years (mean follow-up among survivors 5.6 years), 2626 participants developed cancer and 1095 participants died from cancer. Overall, there was no significant association between CKD category and cancer incidence or death. As compared with the reference group with eGFR ≥75 mL/min/1.73 m^2^, adjusted hazard ratio (HR) estimates for each category of renal function, in descending order, were: 0.98 (95 % CI 0.87–1.10), 0.99 (0.88–1.13), 1.01 (0.84–1.22) and 1.24 (0.97–1.58) for cancer incidence, and 1.03 (95 % CI 0.86–1.24), 0.95 (0.78–1.16), 1.00 (0.76–1.33), and 1.58 (1.09–2.30) for cancer mortality. Among dialysis patients, there was an excess risk of cancers of the urinary tract (adjusted HR: 2.34; 95 % CI 1.10–4.98) and endocrine cancers (11.65; 95 % CI: 1.30–104.12), and an excess risk of death from digestive tract cancers (2.11; 95 % CI: 1.13–3.99), but a reduced risk of prostate cancers (0.38; 95 % CI: 0.18–0.83).

**Conclusions:**

Whilst no association between reduced renal function and the overall risk of cancer was observed, there was evidence among dialysis patients that the risk of cancer was increased (urinary tract, endocrine and digestive tract) or decreased (prostate) at specific sites. Larger studies are needed to characterise these site-specific associations and to identify their pathogenesis.

**Electronic supplementary material:**

The online version of this article (doi:10.1186/s12885-016-2532-6) contains supplementary material, which is available to authorized users.

## Background

The number of people affected by chronic kidney disease (CKD) and end-stage kidney disease (ESKD) is substantial and increasing. The number of new patients with ESKD treated by renal replacement therapy has increased at an average of 8 % per year over the past 10 years globally [1]. Currently, over one million patients are on dialysis worldwide, a number that is estimated to exceed two million over the next decade [2].

CKD is a risk factor for disease affecting other organs. It is well established that people with CKD are at increased risk of developing and dying from cardiovascular disease compared to people without kidney disease [3]. There is also evidence that cancer risk and cancer mortality may be increased in people with CKD, although the associations do appear to be site-specific. It has been reported that reduced renal function is associated with an increased risk of cancers of the kidney or urinary tract [4–7], lip [8], digestive tract [9], lung [4] and some soft tissue and haematological sites [10]. Among dialysis patients, there have also been reports of an increased risk of cervical and possibly thyroid cancers and a reduced risk of prostate cancer [8, 9]. A dose-dependent relationship between albuminuria and bladder or lung cancer risk was observed in a Scandinavian study [11].

Previous observational studies have not examined the extent to which reduced kidney function is associated with increased risk of cancer and cancer death across the full spectrum of kidney disease and in different populations. We hypothesize that reduced kidney function is a risk factor for site-specific cancer and may be a prognostic indicator of poor cancer outcomes. The objective of this study was to determine the overall and site-specific risk for incident cancer and cancer deaths from a broader population of people with CKD, varying from mild to advanced stage disease requiring dialysis.

## Methods

### Study design and participants

Six studies were included in our analysis, of which one was a prospective, population-based cohort study, and five were randomized controlled trials (RCTs). These studies were included because they provided details of serum creatinine, age and gender for the estimation of glomerular filtration rate (GFR), as well as information on site-specific and overall cancer incidence and mortality. Information on non-cancer related mortality was also recorded. All studies were also available to the investigator team for inclusion and so represent a sample of all possible datasets available for analysis.

The cohort study was the Blue Mountains Eye Study (BMES) [12], which included a suburban Australian population aged 49 years or older at baseline (*n* = 3654). The other five RCTs included the Action in Diabetes and Vascular disease: Preterax and Diamicron MR controlled evaluation (ADVANCE) study [13], a multi-centre trial of blood pressure lowering and glucose control in people with type 2 diabetes mellitus (*n* = 11,140); the Perindopril-based blood-pressure-lowering regimen (PROGRESS) study [14], a multi-centre trial of intensive blood pressure lowering using the mixed perindopril and indapamide and placebo in patients with a history of stroke or transient ischaemic attack (*n* = 6105); the Calcium Intake Fracture Outcome (CAIFOS) study [15], a trial of 1500 women that assessed the effects of daily calcium supplements and the risk of osteoporotic fractures in post-menopausal women; the Study of Heart and Renal Protection (SHARP) [16], a multi-centre trial of LDL cholesterol lowering in people with CKD (*n* = 9270) and the Initiating Dialysis Early and Late Study (IDEAL) [17], a trial that compared early and later commencement of dialysis in patients with ESKD (*n* = 828). Full details of each study are reported elsewhere [12–17]. This study involved the use of existing collections of data or records that contain only non-identifiable data. As such, ethics approval was not required according to the National Health and Medical Research Council ethical guidelines on low and negligible risk [18]. Written, informed consent was provided by all participants in each of the studies included in this individual patient meta-analysis.

### Study outcomes

#### Assessment of incident cancers and cancer deaths

Incident cancers were defined as the first cancer diagnosed after inception of the individual studies. Diagnoses of incident cancers and cancer deaths for individual studies were coded according to the *International Classification of Diseases, Ninth and Tenth Revision for cancers (C00 – C96).*

The site-specific cancers were coded as follows: oral cavity and pharynx (C00–C14), digestive (C15–C26), respiratory (C30–C39), bone and cartilage (C40–C41), melanomas (C43), soft tissue/connective tissue (C45–C49), breast (C50), female genital organs (C51–C58), male genital (C60, C62–C63), prostate (C61), urinary tract (C64–C68), central nervous system (C69–C72), endocrine (C73–C75), unknown origin (C76–C80), haematological (C81–C96) and multiple primary sites (C97–C98).

Non-melanocytic skin cancers were excluded from the analyses because they were deemed less clinically important than other cancers and because the Central Cancer Registry of New South Wales and the Western Australia Data Linkage System do not hold information about skin cancers other than melanomas. Information on cancer incidence and mortality in BMES [12] and CAIFOS [15] was obtained from the Central Cancer Registry of New South Wales and the Western Australia Data Linkage System. For all other studies, cancer incidence and mortality were recorded as adverse events during follow-up. Incident cancers and cancer deaths were also categorized into pre-specified groups of similar types to allow site-specific associations to be investigated. Participants known to have been diagnosed with cancer before study commencement were excluded from the analyses.

### Statistical analyses

#### Primary analyses

All statistical analyses were conducted using SAS 9.3. The main analyses of estimated glomerular filtration rate (eGFR) used the Chronic Kidney Disease Epidemiology Collaboration (CKD-EPI) equation, but they were repeated using the four-variable MDRD equation [19, 20]. The relevance of baseline eGFR to the risk of cancer incidence and cancer mortality was estimated using Cox proportional hazards regression models stratified by study. All regression analyses were adjusted for age, sex, ethnicity and smoking status. The shapes of the association between baseline renal function and cancer risk and deaths were assessed by grouping participants into five categories defined by their baseline eGFR (eGFR ≥75; ≥60 to <75; ≥45 to <60; <45 ml/min/1.73 m^2^ but not on dialysis; and on dialysis). Relative risks, estimated by the hazard ratios from the Cox regression models, are presented graphically with a group-specific confidence interval (CI) derived only from the variance of the log risk in that one category. Each relative risk, including that for the reference group, is associated with a group-specific CI that can be thought of as reflecting the amount of data only in that one category which, if desired, allows for an appropriate statistical comparison to be made between any two groups [21]. Throughout the text, all quoted relative risks are provided with the CI for the comparison with the specified reference group. Analyses were repeated separately for men and women and also for specific common groupings of cancer sites. To assess the extent to which the observed associations may be the result of reverse causality, the primary analyses were repeated excluding cancers and cancer deaths that occurred within the first 2 years of follow-up. Finally, the potential relevance of the competing risk of non-cancer related death was considered using a stratified proportional sub-distribution hazard model [22].

## Results

### Baseline characteristics of participants

Among the 33,680 participants in the six studies, 1236 (3.6 %) were excluded because of missing values for age, gender or eGFR and a further 387 (1.1 %) were excluded because of a prior history of cancer, leaving a total of 32,057 participants. Of these, 18,427 (57.5 %) were men, 15,429 (48.1 %) were previous or current smokers and 22,263 (69.4 %) were of white race (Table 1 and Additional file 1). A total of 9594 (29.9 %) participants had eGFR ≥75 ml per min per 1.73 m^2^; 6681 (20.8 %) had an eGFR of at least 60 but less than 75 ml per min per 1.73 m^2^; 4931 (15.4 %) had an eGFR of at least 45 but less than 60 ml per min per 1.73 m^2^; 7828 (24.4 %) had an eGFR less than 45 per min per 1.73 m^2^ and 3023 (9.4 %) participants were on dialysis (Table 2). All participants on dialysis were from SHARP [16].

**Table 1:** Baseline characteristics of 32057 eligible participants, by CKD status

	CKD status (CKD EPI-estimated GFR (mL/min/1.73 m^2^))				Dialysis (*n* = 3023)
	Greater than 75 (*n* = 9594)	60 to 75 (*n* = 6681)	45 to 60 (*n* = 4931)	Less than 45 (*n* = 7828)	
Age at baseline (years)	63 (8)	67 (8)	70 (9)	65 (12)	60 (12)
Male	5983 (62 %)	3739 (56 %)	2266 (46 %)	4521 (58 %)	1918 (63 %)
Ethnicity					
White	5570 (58 %)	4985 (75 %)	3801 (77 %)	5744 (73 %)	2163 (72 %)
Asian	3847 (40 %)	1555 (23 %)	1035 (21 %)	1701 (22 %)	564 (19 %)
Other/not specified	177 (2 %)	141 (2 %)	95 (2 %)	381 (5 %)	298 (10 %)
Higher education	110 (1 %)	605 (9 %)	886 (18 %)	1835 (23 %)	660 (22 %)
Ever smoked	4683 (49 %)	3179 (48 %)	2221 (45 %)	3841 (49 %)	1505 (50 %)
Body mass index (kg/m^2^)	27.2 (4.9)	27.3 (4.8)	27.2 (4.9)	27.5 (5.5)	26.5 (5.9)
Systolic blood pressure (mm Hg)	144 (25)	145 (21)	146 (21)	142 (22)	138 (24)
Diastolic blood pressure (mm Hg)	83 (20)	82 (11)	82 (11)	80 (12)	78 (13)
MDRD-estimated GFR (mL/min/1.73 m^2^)	94.4 (22.0)	68.7 (4.5)	55.5 (4.5)	25.7 (12.2)	-
CKD EPI-estimated GFR (mL/min/1.73 m^2^)	89.1 (9.6)	67.4 (4.3)	53.4 (4.2)	24.3 (11.7)	-
Total cholesterol (mg/dL)	203 (44)	210 (47)	215 (48)	196 (48)	179 (45)
Triglycerides (mg/dL)	170 (121)	169 (115)	176 (113)	202 (136)	205 (164)
Follow-up time (years)	5.0 (4.4–5.1)	5.0 (4.4–5.5)	5.0 (4.4–10.4)	4.5 (3.9–54)	4.4 (3.2–5.4)

Mean (SD), median (IQR) or n (%) shown

**Table 2:** Number of incident cancers (annual rate per 1000 patients) by CKD status and cancer site

	CKD status (CKD EPI-estimated GFR (mL/min/1.73 m^2^))				Dialysis (*n* = 3023)	All (*n* = 32057)
	Greater than 75 (*n* = 9594)	60 to 75 (n = 6681)	45 to 60 (*n* = 4931)	Less than 45 (*n* = 7828)		
Total person years	48216	41013	34630	35011	11948	170819
All sites	596 (13.9)	621 (14.0)	580 (15.7)	619 (18.5)	210 (22.2)	2626 (15.4)
Oral cavity and pharynx	12 (0.3)	12 (0.3)	12 (0.3)	11 (0.3)	9 (0.6)	56 (0.3)
Digestive	172 (4.0)	177 (3.8)	156 (4.0)	147 (4.3)	54 (6.6)	706 (4.1)
Respiratory	88 (1.9)	86 (2.0)	59 (1.7)	63 (1.9)	26 (2.9)	322 (1.9)
Melanomas	16 (0.4)	24 (0.5)	43 (1.2)	40 (1.2)	7 (0.8)	130 (0.8)
Breast	60 (1.4)	69 (1.8)	74 (1.7)	60 (1.7)	14 (1.2)	277 (1.6)
Female genital	17 (0.4)	20 (0.4)	19 (0.7)	23 (0.7)	8 (0.9)	87 (0.5)
Prostate	79 (1.7)	80 (1.9)	72 (2.1)	85 (2.8)	16 (1.9)	332 (1.9)
Male genital	1 (0.0)	0 (0.0)	1 (0.0)	2 (0.1)	1 (0.1)	5 (0.0)
Soft tissue/connective tissue	3 (0.1)	3 (0.1)	5 (0.1)	12 (0.4)	0 (0.0)	23 (0.1)
Urinary tract	40 (1.0)	30 (0.7)	35 (1.3)	83 (2.5)	40 (4.1)	228 (1.3)
Central nervous system	11 (0.3)	15 (0.3)	9 (0.2)	8 (0.2)	2 (0.3)	45 (0.3)
Endocrine	5 (0.1)	2 (0.1)	5 (0.2)	5 (0.2)	12 (0.7)	29 (0.2)
Haematological	47 (1.0)	53 (1.1)	43 (1.0)	42 (1.2)	16 (1.5)	201 (1.2)
Multiple primary sites	5 (0.2)	14 (0.3)	14 (0.3)	9 (0.2)	0 (0.0)	42 (0.2)
Unknown origin	17 (0.4)	10 (0.2)	10 (0.2)	18 (0.6)	5 (0.7)	60 (0.4)
Bone and cartilage	2 (0.0)	0 (0.0)	0 (0.0)	0 (0.0)	0 (0.0)	2 (0.0)
Others	8 (0.2)	10 (0.3)	7 (0.3)	0 (0.0)	0 (0.0)	25 (0.1)
Site data not available	13	16	16	11	0	56

Rates in CKD status group directly standardized for age sex, using 10-year age intervals

### Incidence of cancer and deaths from cancer

During an average follow-up (among survivors) of 5.6 years, 2626 participants developed cancer (average incidence rate 15.4 per 1000 person-years [py]; Table 2) and 1095 died from cancer (6.2 per 1000 py; Table 3). Cancers of the digestive system (*n* = 706; 4.1 per 1000 py) were the most common cancers, followed by prostate cancers (*n* = 332; 1.9 per 1000 py), cancers of the respiratory system (*n* = 322; 1.9 per 1000 py), breast cancers (*n* = 277; 1.6 per 1000 py), and cancers of the urinary tract (n = 228; 1.3 per 1000 py). Cancers of the digestive system were also the most common cause of cancer death (*n* = 373; 2.1 per 1000 py), followed by cancers of the respiratory tract (*n* = 249; 1.4 per 1000 py).

**Table 3:** Number of cancer deaths (annual rate per 1000 patients) by CKD status and cancer site

	CKD status (CKD EPI-estimated GFR (mL/min/1.73 m^2^))				Dialysis (*n* = 3023)	All (*n* = 32057)
	Greater than 75 (*n* = 9594)	60 to 75 (*n* = 6681)	45 to 60 (*n* = 4931)	Less than 45 (*n* = 7828)		
Total person years	49403	42615	36452	36538	12436	177443
All sites	240 (5.8)	272 (5.7)	247 (5.9)	246 (7.0)	90 (10.8)	1095 (6.2)
Oral cavity and pharynx	6 (0.2)	2 (0.0)	3 (0.1)	4 (0.1)	4 (0.2)	19 (0.1)
Digestive	84 (2.0)	94 (1.9)	78 (1.9)	81 (2.3)	36 (4.2)	373 (2.1)
Respiratory	71 (1.5)	67 (1.5)	44 (1.2)	47 (1.4)	20 (2.4)	249 (1.4)
Melanomas	3 (0.1)	3 (0.1)	8 (0.2)	6 (0.2)	5 (0.7)	25 (0.1)
Breast	5 (0.2)	10 (0.2)	6 (0.1)	11 (0.2)	0 (0.0)	32 (0.2)
Female genital	5 (0.2)	7 (0.1)	13 (0.3)	6 (0.2)	2 (0.3)	33 (0.2)
Prostate	9 (0.3)	13 (0.3)	22 (0.5)	11 (0.3)	1 (0.1)	56 (0.3)
Male genital	0 (0.0)	0 (0.0)	0 (0.0)	0 (0.0)	0 (0.0)	0 (0.0)
Soft tissue/connective tissue	1 (0.0)	0 (0.0)	6 (0.1)	8 (0.3)	1 (0.1)	16 (0.1)
Urinary tract	10 (0.2)	7 (0.1)	7 (0.3)	18 (0.5)	5 (0.8)	47 (0.3)
Central nervous system	9 (0.2)	12 (0.2)	10 (0.2)	7 (0.2)	2 (0.3)	40 (0.2)
Endocrine	1 (0.0)	1 (0.0)	0 (0.0)	1 (0.0)	2 (0.3)	5 (0.0)
Haematological	14 (0.3)	29 (0.6)	26 (0.5)	20 (0.5)	7 (0.8)	96 (0.5)
Multiple primary sites	6 (0.2)	13 (0.3)	10 (0.2)	6 (0.1)	0 (0.0)	35 (0.2)
Unknown origin	11 (0.2)	12 (0.2)	11 (0.3)	16 (0.5)	5 (0.7)	55 (0.3)
Bone and cartilage	0 (0.0)	0 (0.0)	0 (0.0)	0 (0.0)	0 (0.0)	0 (0.0)
Others	3 (0.0)	0 (0.0)	1 (0.1)	0 (0.0)	0 (0.0)	4 (0.0)
Site data not available	2	2	2	4	0	10

Rates in CKD status group directly standardized for age sex, using 10-year age intervals

### Relevance of renal function to cancer incidence and cancer death

**Fig. 1:**
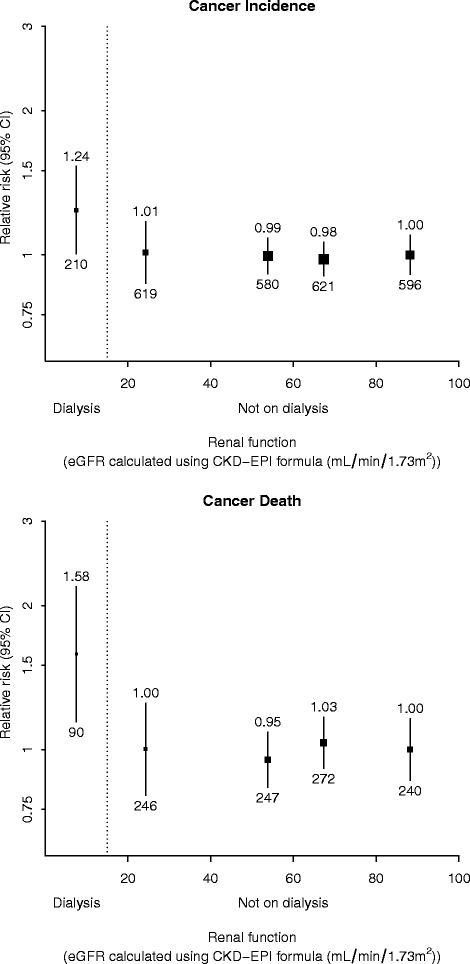
Relevance of renal function to cancer incidence and cancer death after adjustment for age, sex, ethnicity and smoking status. Relative risks are stated above 95 % CI and the number of events is given below 95 % CI

Overall, there was no significant association between baseline stage of kidney disease and cancer incidence or cancer mortality. For cancer incidence, compared with the reference category with eGFR ≥75 mL/min/1.73 m^2^, adjusted hazard ratio (HR) estimates for the other renal function categories, in order of declining renal function, were 0.98 (95 % CI 0.87–1.10), 0.99 (0.88–1.13), 1.01 (0.84–1.22) and 1.24 (0.97–1.58) respectively (Fig. 1). For cancer death, these four estimates were 1.03 (95 % CI 0.86–1.24), 0.95 (0.78–1.16), 1.00 (0.76–1.33) and 1.58 (1.09–2.30) respectively. Estimates were largely unaltered after exclusion of the first 2 years’ follow-up 1.12 (95 % CI 0.97–1.29), 1.11 (0.95–1.30), 1.15 (0.92–1.44), 1.32 (0.97–1.81) for cancer incidence; 1.12 (0.91–1.38), 1.03 (0.82–1.29), 1.06 (0.77–1.47) and 1.78 (1.14–2.77) for cancer death (Additional file 2).

The association between baseline category of renal function and cancer incidence and cancer death was also unaffected by adjustment for competing risks from non-cancer death, although the relative increase in cancer death seen for dialysis patients was attenuated (Additional file 3). Compared to participants with eGFR ≥75 ml/min per 1.73 m ^2^, the adjusted HRs for cancer incidence in descending order of renal function category were 1.00 (95 % CI 0.89–1.12), 1.01 (0.89–1.15), 0.95 (0.79–1.15) and 1.03 (0.80–1.31), while for cancer death the estimates were 1.06 (0.89–1.27), 0.98 (0.80–1.20), 0.93 (0.70–1.24) and 1.25 (0.86–1.82) for participants on dialysis.

**Fig. 2:**
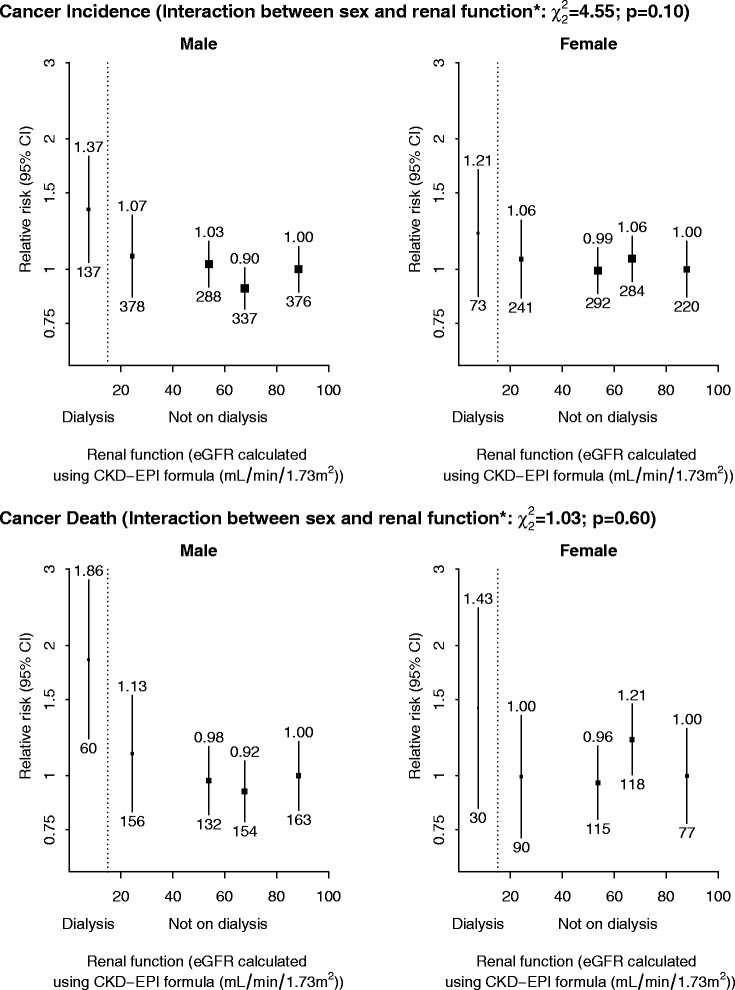
Sex-specific relevance of renal function to cancer incidence and cancer death after adjustment for age, ethnicity and smoking status. Relative risks are stated above 95 % CI and the number of events is given below 95 % CI. *Joint test of the significance of two interaction terms (between sex and, respectively, a linear and quadratic term for ordered renal function group) done by comparing the difference in -2 log L between the two nested models

There was no significant association in either sex between renal function and cancer incidence or cancer mortality, nor did the overall associations differ by gender (test for interaction between gender and renal function *p* = 0.10 for incident cancer; *p* = 0.60 for cancer death; Fig. 2).

### Relevance of renal function to site-specific cancer risk

**Fig. 3:**
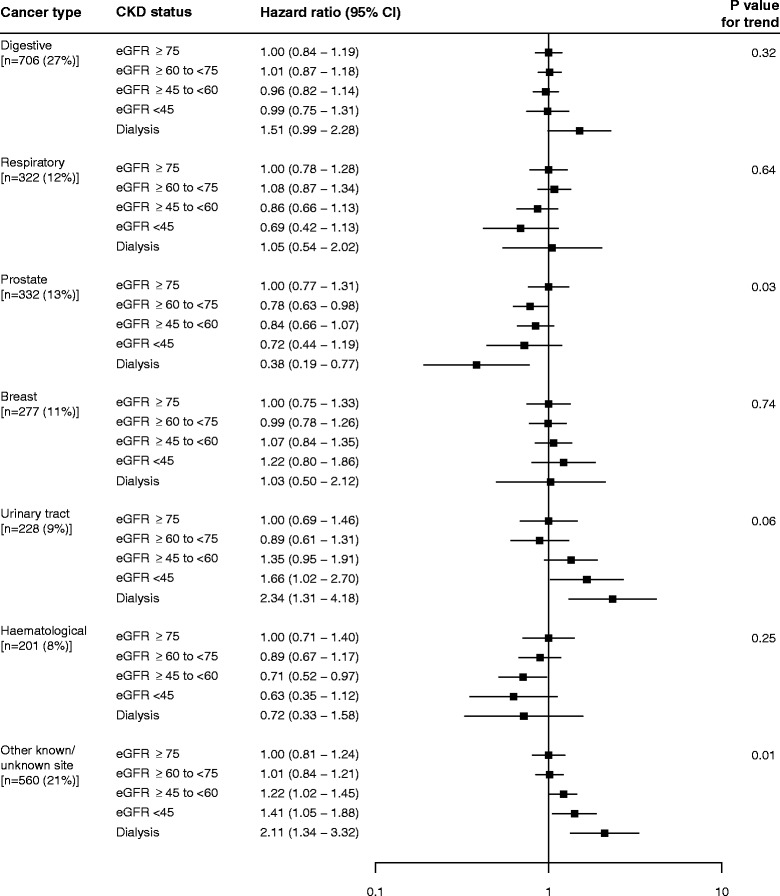
Relevance of renal function to site specific cancer incidence after adjustment for age, sex, ethnicity and smoking status

Associations between baseline category of renal function and cancer risk were observed for specific cancer sites (Fig. 3). With declining renal function, there was a non-significant trend (*p* = 0.06, Fig. 3) towards an increased risk of urinary tract cancer, with an increased risk of such cancers among dialysis patients as compared with participants with eGFR ≥75 ml per min per 1.73 m^2^ (adjusted HR 2.34 [95 % CI: 1.10–4.97]). There was also a significant trend towards an increased risk of other known/unknown cancers (trend *p* = 0.01), which appeared to be chiefly attributable to an increased risk of endocrine (mostly thyroid) cancers, with an increased risk of endocrine cancers among dialysis patients as compared to participants with eGFR ≥75 ml per min per 1.73 m^2^ (adjusted HR 11.65, 95 % CI 1.30–104.12; Additional file 4). With declining renal function there was also a significant trend towards reduced risk of prostate cancer (trend *p* = 0.03, Fig. 3). In addition, dialysis patients had a twofold higher risk of death from cancers of the digestive tract (adjusted HR: 2.11; 95 % CI: 1.13–3.99), however the excess in digestive cancer incidence did not reach statistical significance (HR 1.51, 95 % CI 0.94–2.42).

## Discussion

We analysed individual patient data from six prospective studies of 32,057 participants with various levels of renal function, followed for an average of 5 years. Although in the pre-specified analyses there was no significant association between renal impairment and the overall risk of cancer or of cancer death, several notable findings emerged when these findings were examined in greater detail. First, as compared with people with eGFR ≥75 ml/min/1.73 m^2^, patients on dialysis had a non-significant excess risk of any cancer (HR 1.24, 95 % CI 0.97–1.58) together with a statistically significant increase in the risk of cancer death (HR 1.58, 95 % CI 1.09–2.30). Second, closer inspection of data on cancer risk in particular sites indicated that the lack of any overall association masked clear associations for specific cancer types: in particular, with declining renal function there were trends towards increased risks of urinary tract and endocrine (mostly thyroid) cancers but also lower risk of prostate cancer. Taken together, our findings extend previous reports of associations between renal function and cancer risk to people with a wider degree of renal dysfunction, and our findings for particular cancer sites are consistent with these earlier reports [4, 5, 7, 8].

It is well known that the lifespan of people on dialysis is reduced as a consequence of premature death from both cardiovascular and non-cardiovascular causes [23–25]. Our study findings suggest that cancer is a contributing factor to the increased risk of non-vascular death among patients on dialysis. A 1.5-fold increase in the risk of cancer death is broadly consistent with previous observational studies that have reported an excess risk of cancer in the range of 1.2 and 1.4-fold among those on dialysis using registry analyses [8, 10], but our findings make clear that the magnitude of any relative excess of cancer in a given dialysis population will be determined by the relative frequency of different cancers which, depending on the subtype, may be associated (positively or negatively) or unassociated with declining renal function. The distribution of cancer types will in turn depend on the gender, age, and ethnicity of the population, as well as other factors.

Some previous studies have reported an association between renal function and any cancer [4, 8] whilst others did not [5, 9]. For dialysis patients, other studies have reported an increased risk of cancer, especially of the kidney and urinary tract [8, 9] but also of thyroid cancer [8] and some digestive tract cancers [8, 9]. Previous studies have also shown that the risk of prostate cancer is reduced among dialysis patients [9]. In contrast to previous studies [9] we did not observe an increased risk of oral cavity, respiratory or haematological cancers among those with reduced kidney function. Moreover, whilst a previous study suggested that women on dialysis were at increased risk of cervical cancer [8, 9], we did not observe a significantly higher risk of female genital cancers for dialysis patients. This apparent heterogeneity of the available literature is consistent with the observation that associations between declining renal function and cancer risk are dependent on cancer subtype, which may vary between different study populations, and it implies that studies (or meta-analyses of studies) involving much larger numbers of cancers with detailed subtyping information are needed to gain a better understanding of these associations.

The present study adds to the current evidence that the excess cancer observed in people on dialysis may not be driven solely by viral carcinogenesis as previously suggested [8], but could also be influenced by the uraemic milieu associated with severe renal dysfunction. Uraemia is often characterized as a state of immune dysfunction. The different types of uraemic toxins may exert antagonistic interactions of pro-inflammatory and immunosuppressive responses, leading to increased risks of infections and malignancy [26]. In addition, people on dialysis retain solutes, which may impair the anti-tumour activity of certain immune cell types such as natural killer and dendritic cells, promote angiogenesis and enhance accelerated growth of aggressive tumours [26]. Future studies that explore the relationship between impaired renal function and risk for particular cancer subtypes (rather than for cancer of all types) may be able to provide a better understanding of these processes.

Our study has several strengths. The present meta-analysis represents one of the largest cohorts of individuals with diverse patient characteristics to have examined the effects of reduced kidney function and risk of cancer and cancer death. The availability of individual data allowed for an assessment of the potential influence on estimates of competing risks and reverse causality bias. There are also some potential limitations. First, we may not have had sufficient follow-up time to reliably detect a small but significant effect among those with moderate stage CKD, particularly for cancers such as colorectal, breast and prostate cancer which have a long latency period relative to the period of observation in the included studies. Second, our study was not powered to detect a statistically significant interaction between gender and the effects of reduced kidney function on cancer incidence and death, or to reliably investigate the relevance of renal function to site specific cancer risk. Third, none of the included studies considered cancer as their primary outcome, so cancer reports may not have been confirmed, for example, by pathology reports. The reliability of the cancer outcomes may also have varied between the individual studies. In general, cancer incidence and mortality data were recorded by the treating physicians who confirmed the cancer diagnoses and/or deaths. It is likely that systematic coding errors may have occurred for the different studies and resulted in over or under-estimation of the causes and/or the potential missing causes of death. Fourth, only one study (SHARP) contributed data evaluating the link between dialysis and cancer, whereas all studies contributed data for earlier stage CKD. Finally, while adjustments were made for potential confounders, residual confounding from unmeasured factors may exist.

## Conclusion

In summary, this study indicates that reduced renal function is associated with an increased risk of urinary tract, digestive tract and thyroid cancers, but also with a reduced risk of prostate cancer in men. The risk is most marked among dialysis patients, but our study did not have sufficient power to exclude an increase in risk of particular cancers among patients with less severe renal impairment. Much larger studies are needed to facilitate an understanding of the association between renal function and the risk of specific cancers, and to identify possible mechanisms through which renal impairment may modulate cancer risk.

## Abbreviations

ADVANCE, action in diabetes and vascular disease: preterax and diamicron mr controlled evaluation study; BMES, blue mountains eye study; CAIFOS, calcium intake fracture outcome study; CI, confidence interval; CKD, chronic kidney disease; CKD-EPI, chronic kidney disease epidemiology collaboration; eGFR, estimated glomerular filtration rate; ESKD, end stage kidney disease; HR, hazard ratio; PROGRESS, perindopril-based blood-pressure-lowering regimen study; RCTs, randomised controlled trials; SHARP, study of heart and renal protection

## Additional files

Additional file 1: Baseline characteristics of 32057 eligible participants, by study. (PDF 160 kb) Additional file 2: Relevance of renal function to cancer incidence and cancer death, after adjustment for age, sex, ethnicity and smoking status, excluding events in the first 2-years of follow-up. (PDF 9 kb) Additional file 3: Relevance of renal function to cancer incidence and cancer death, after adjustment for age, sex, ethnicity and smoking status, using Fine and Gray regression. (PDF 9 kb) Additional file 4: Relevance of renal function to site specific cancer incidence after adjustment for age, sex, ethnicity and smoking status. (PDF 157 kb)
