# Supplementary material for: Chronic kidney disease and the risk of cancer: an individual patient data meta-analysis of 32,057 participants from six prospective studies
Source: BMC Cancer. 2016 Jul 16;16:488. doi: 10.1186/s12885-016-2532-6 (PMC4947287; doi:10.1186/s12885-016-2532-6)
Supplement: Additional file 1: — Baseline characteristics of 32057 eligible participants, by study. (PDF 160 kb) [file 12885_2016_2532_MOESM1_ESM.pdf]

Additional file 1: Baseline characteristics of 32057 eligible participants, by study

	ADVANCE (n=11096)	PROGRESS (n=6001)	SHARP (n=9052)	BMES (n=3882)	IDEAL (n=828)	CAIFOS (n=1198)
Age at baseline (years)	66 (6)	64 (10)	62 (12)	71 (10)	60 (12)	75 (3)
Male	6386 (58%)	4173 (70%)	5657 (62%)	1669 (43%)	542 (65%)	0 (0%)
Ethnicity						
White	6643 (60%)	3568 (59%)	6484 (72%)	3778 (97%)	592 (71%)	1198 (100%)
Asian	4240 (38%)	2326 (39%)	2047 (23%)	16 (<1%)	73 (9%)	0 (0%)
Other/not specified	213 (2%)	107 (2%)	521 (6%)	88 (2%)	163 (20%)	0 (0%)
Higher education	-	-	2271 (25%)	1677 (43%)	148 (18%)	-
Ever smoked	4653 (42%)	3462 (58%)	4415 (49%)	1968 (51%)	498 (60%)	433 (36%)
Body mass index (kg/m²)	28.3 (5.2)	25.7 (3.8)	27.1 (5.6)	26.6 (4.7)	29.0 (6.0)	27.1 (4.6)
Systolic blood pressure (mm Hg)	145 (22)	147 (19)	139 (22)	145 (32)	143 (21)	138 (18)
Diastolic blood pressure (mm Hg)	81 (11)	86 (11)	79 (13)	85 (27)	79 (11)	73 (11)
MDRD-estimated GFR (mL/min/1.73m²)	78.1 (24.7)	77.8 (23.7)	26.6 (13.0)	65.6 (17.4)	9.8 (2.3)	69.5 (14.4)
CKD EPI-estimated GFR (mL/min/1.73m²)	74.5 (17.6)	74.6 (18.3)	25.4 (13.0)	63.1 (16.8)	8.9 (1.9)	66.6 (13.3)
Total cholesterol (mg/dL)	201 (46)	-	189 (46)	229 (42)	174 (50)	227 (42)
Triglycerides (mg/dL)	173 (114)	-	206 (152)	173 (112)	193 (157)	139 (65)
On dialysis	0 (0%)	0 (0%)	3025 (33%)	0 (0%)	0 (0%)	0 (0%)
Follow-up time (years)	5.0 (4.7-5.0)	4.0 (3.6-4.4)	4.4 (3.9-5.4)	14.6 (8.8-16.0)	4.2 (2.8-5.7)	10.3 (10.1-10.5)

Mean (SD), median (IQR) or n (%) shown.
