# Supplementary material for: Chronic kidney disease and the risk of cancer: an individual patient data meta-analysis of 32,057 participants from six prospective studies
Source: BMC Cancer. 2016 Jul 16;16:488. doi: 10.1186/s12885-016-2532-6 (PMC4947287; doi:10.1186/s12885-016-2532-6)
Supplement: Additional file 2: — Relevance of renal function to cancer incidence and cancer death, after adjustment for age, sex, ethnicity and smoking status, excluding events in the first 2-years of follow-up. (PDF 9 kb) [file 12885_2016_2532_MOESM2_ESM.pdf]

Additional file 3: Relevance of renal function to cancer incidence and death, after adjustment for age, sex, ethnicity and smoking status, excluding events in the first 2 years of follow−up

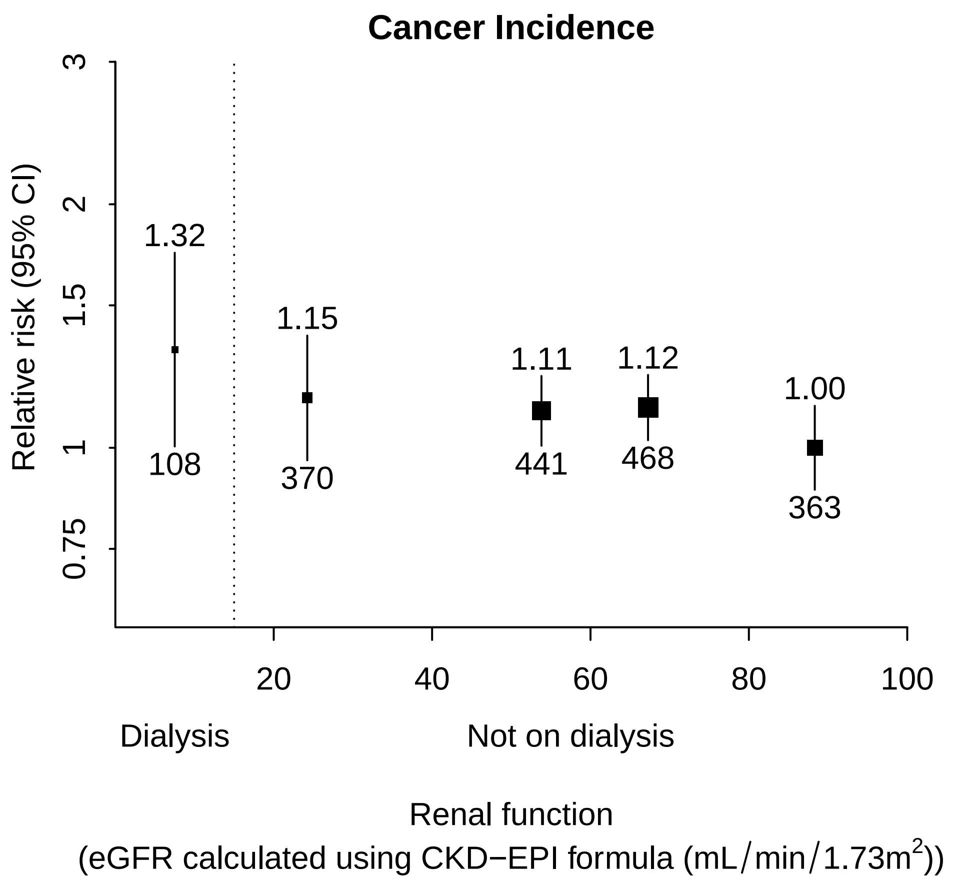

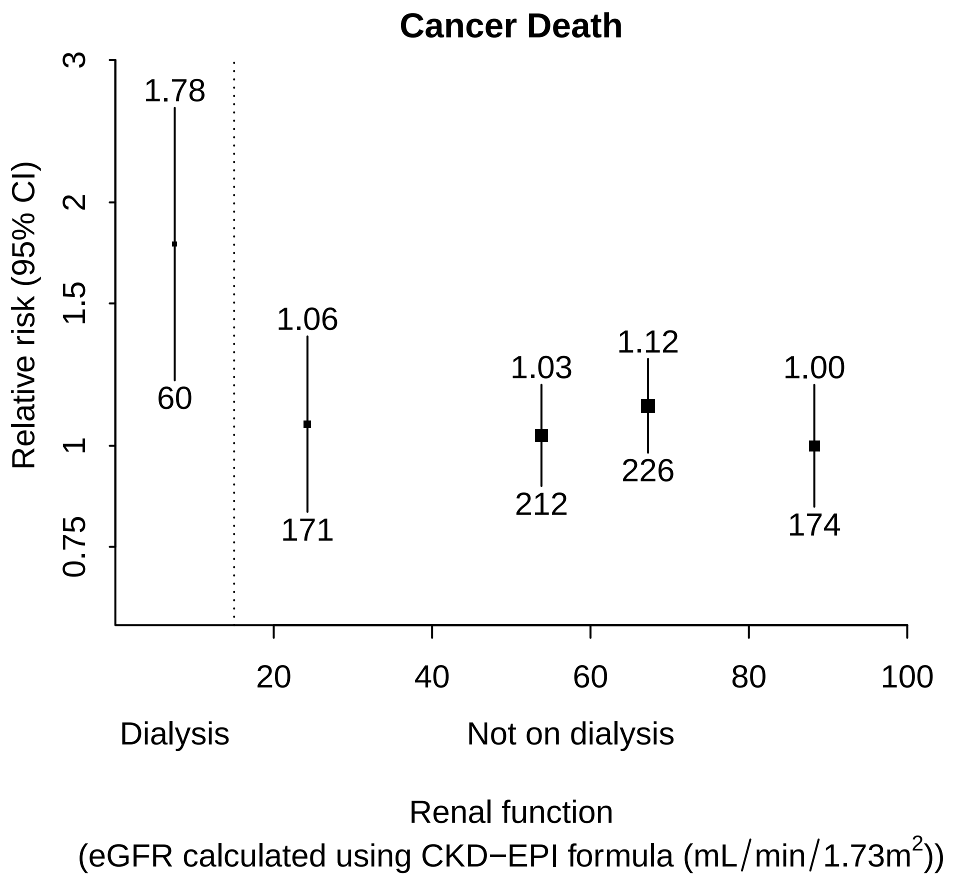

Relative risks are stated above 95% CI and the number of events is given below 95% CI.
