# Supplementary material for: Chronic kidney disease and the risk of cancer: an individual patient data meta-analysis of 32,057 participants from six prospective studies
Source: BMC Cancer. 2016 Jul 16;16:488. doi: 10.1186/s12885-016-2532-6 (PMC4947287; doi:10.1186/s12885-016-2532-6)
Supplement: Additional file 4: — Relevance of renal function to site specific cancer incidence after adjustment for age, sex, ethnicity and smoking status. (PDF 157 kb) [file 12885_2016_2532_MOESM4_ESM.pdf]

## Additional file 2: Relevance of renal funtion to site specific cancer incidence after adjustment for age, sex, ethnicity and smoking status

| | Hazard ratio (95% Confidence Interval) by CKD status (CKD EPI-estimated GFR (mL/min/1.73m²)) | | | | | |
|---|---|---|---|---|---|---|
| | **Greater than 75 (n=9594)** | **60 to 75 (n=6681)** | **45 to 60 (n=4931)** | **Less than 45 (n=8808)** | **Dialysis (n=3023)** | **P value for trend across groups** |
| Oral cavity and pharynx | 1.00 | 1.04 (0.45 - 2.41) | 1.17 (0.48 - 2.90) | 1.09 (0.28 - 4.21) | 2.57 (0.53 - 12.46) | 0.44 |
| Digestive† | 1.00 | 1.01 (0.82 - 1.26) | 0.96 (0.76 - 1.23) | 0.99 (0.70 - 1.40) | 1.51 (0.94 - 2.42) | 0.32 |
| Respiratory† | 1.00 | 1.08 (0.79 - 1.47) | 0.86 (0.60 - 1.24) | 0.69 (0.39 - 1.23) | 1.05 (0.51 - 2.18) | 0.64 |
| Melanomas | 1.00 | 0.93 (0.48 - 1.78) | 1.71 (0.92 - 3.20) | 1.60 (0.66 - 3.85) | 1.86 (0.54 - 6.44) | 0.23 |
| Breast† | 1.00 | 0.99 (0.69 - 1.42) | 1.07 (0.73 - 1.54) | 1.22 (0.72 - 2.07) | 1.03 (0.46 - 2.30) | 0.74 |
| Female genital | 1.00 | 0.98 (0.50 - 1.90) | 0.88 (0.43 - 1.79) | 1.49 (0.60 - 3.66) | 1.73 (0.51 - 5.89) | 0.46 |
| Prostate† | 1.00 | 0.78 (0.56 - 1.09) | 0.84 (0.59 - 1.21) | 0.72 (0.40 - 1.30) | 0.38 (0.18 - 0.83) | 0.03 |
| Soft tissue/connective tissue | 1.00 | 0.96 (0.19 - 4.83) | 2.16 (0.48 - 9.68) | 5.62 (1.07 - 29.62) | -* | 0.11 |
| Urinary tract† | 1.00 | 0.89 (0.55 - 1.44) | 1.35 (0.83 - 2.19) | 1.66 (0.85 - 3.24) | 2.34 (1.10 - 4.97) | 0.06 |
| Central nervous system | 1.00 | 1.02 (0.45 - 2.29) | 0.60 (0.22 - 1.59) | 0.74 (0.19 - 2.93) | 0.87 (0.10 - 7.49) | 0.71 |
| Endocrine | 1.00 | 0.51 (0.10 - 2.67) | 1.82 (0.49 - 6.75) | 2.13 (0.30 - 15.07) | 11.65 (1.30 - 104.12) | 0.12 |
| Haematological† | 1.00 | 0.89 (0.59 - 1.34) | 0.71 (0.45 - 1.13) | 0.63 (0.31 - 1.27) | 0.72 (0.29 - 1.76) | 0.25 |

†Results also presented in Figure 3. *There were no incident cancers of type soft tissue/connective tissue observed for those on dialysis, so a HR could not be estimated.
